# Assessing the Opportunities of Spectral Shaping by Quantum Cutting for Perovskite/Silicon Tandem Solar Cells

**DOI:** 10.1021/acsenergylett.6c00270

**Published:** 2026-04-17

**Authors:** Brian M. Wieliczka, Jakob Möbs, Nakita K. Noel, Henry J. Snaith

**Affiliations:** † Clarendon Laboratory, Department of Physics, 6396 University of Oxford, Oxford OX1 3PU, United Kingdom; ‡ Institute for Inorganic and Analytical Chemistry, Justus-Liebig-University Gießen, D-35392 Gießen, Germany; § Center for Materials Research (LAMA), Justus-Liebig-University Gießen, Heinrich-Buff-Ring 16, 35392 Gießen, Germany

## Abstract

Quantum cutting using Yb-doped halide perovskites provides a promising route for reshaping the solar spectrum for perovskite/silicon tandem photovoltaics by converting one high-energy ultraviolet or visible photon into two near-infrared photons. Using detailed balance analysis, we find that the idealized efficiency limit of perovskite/silicon tandem solar cells remains largely unchanged but identify several opportunities for using a quantum cutting layer. Integrating such a layer shifts the optimal top cell bandgap from 1.7 to ∼1.45 eV, opening the possibility of using potentially more stable, neat iodide perovskite compositions without sacrificing performance. Additionally, beyond efficiency, quantum cutting layers could mitigate ultraviolet-induced degradation of the solar cell stack. Finally, we identify key areas of research needed to unlock this spectral reshaping strategy.

## Graphical abstract

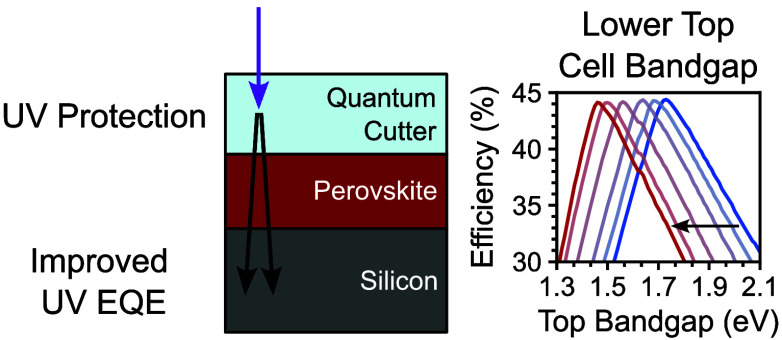

Quantum cutting emitters are reported to convert a single high-energy ultraviolet (UV) or visible photon into two low-energy near-infrared (NIR) photons.^1–5^ Adding a quantum cutting layer with a photoluminescence quantum yield (PLQY) of >100% to a single junction silicon solar cell promises to significantly improve its power conversion efficiency (PCE).^6,7^ However, perovskite/silicon tandem solar cells are touted as the next major step in photovoltaics by many academic and industry researchers and are already delivering efficiencies well beyond the thermodynamic limit for a single-junction solar cell.^8–11^ In this perspective, we consider the addition of a quantum cutting layer to a perovskite/silicon tandem solar cell and use detailed balance analysis to analyze the theoretical efficiency limit with photon multiplication via quantum cutting. Surprisingly, we find that quantum cutting does not substantially increase the fundamental limits to power conversion efficiency, but creates several unique opportunities for tandems, particularly with respect to stability. Reshaping the solar spectrum using a quantum cutting layer reduces the photocurrent in the top cell and increases the photocurrent in the silicon cell, shifting the optimal top cell bandgap for a perovskite/silicon tandem solar cell to lower energy. This shift could enable the use of neat iodide perovskites, sidestepping the stability challenges of halide segregation.^12^ Additionally, the quantum cutting layer could protect the underlying perovskite device stack from UV-induced degradation by acting as a UV filter while still harvesting this power.^13^ We then compare these idealized detailed balance analysis calculations to current state-of-the-art tandem solar cells to identify ways in which the use of quantum cutting layers can result in improved efficiency and stability of these devices. Finally, we identify key areas in the literature which would, in our opinion, require further investigation to enable the successful incorporation of a quantum cutting layer into a highly efficient and stable perovskite/silicon tandem solar cell.

Quantum cutting has been reported in a range of materials, but the most promising candidate for solar energy conversion is cesium lead halide doped with Yb^3+^ (Yb:CsPb­(Cl_1–x_Br_*x*_)_3_). Quantum cutting was first theorized in 1957 by Dexter. In this process, a sensitizer (an ion, atom, or group of ions/atoms) excited by absorption of a photon can transfer its energy to two activators (also ions, atoms, or groups), thus creating two excited states.^14^ The activators can subsequently emit a single photon each, allowing a PLQY greater than unity, distinguishing themselves from down converters limited to ≤100% PLQY.^15^ As of the beginning of the 21st century, a number of quantum cutting materials targeting the conversion of UV into visible light for display applications had already been developed. These materials were primarily based on the emission from various lanthanides doped into metal halides,^16–23^ metal oxides,^24–26^ glasses,^27–29^ and glass ceramics.^30,31^ However, these materials are inadequate for use in solar energy conversion due to their weak and narrow absorption which relies on a parity-forbidden 4*f* → 4*f*-transition in lanthanide ions. They also often require coupling between multiple lanthanide codopants. Halide perovskites emerged as a host matrix for Yb^3+^, offering a seemingly ideal solution for use in photovoltaics.^1,2,5^ First, the Yb^3+^-ion is the near ideal emitter, with emitted photon energy at 1.25 eV, which fits well with the 1.12 eV bandgap of silicon.^32^ Second, the absorption of the CsPb­(Cl_1–x_Br_*x*_)_3_ host lattice is strong and broad with a tunable absorption onset based on the halide composition.^5,7,33^ Milstein et al. found that the bandgap of the quantum cutting matrix (QC_gap_) strongly influenced the PLQY, with a threshold of >100% PLQY for QC_gap_ values just over twice the Yb^3+^ emission energy, approximately 2.53 eV.^33^ Last, unlike previously developed quantum cutters, lanthanide codopants are not necessary for quantum cutting in Yb^3+^-doped perovskites. Absolute PLQY values of up to 190% have been reported for Yb:CsPb­(Cl_1–*x*_Br_*x*_)_3_ nanocrystal solutions and thin films.^1–4,33–36^

## Detailed Balance Analysis

Although quantum cutting appears to be a very realistic approach for increasing the efficiency of silicon photovoltaic cells, the estimated maximum efficiencies of such devices remain lower than those of two-junction, perovskite/silicon tandem solar cells. However, the impact of, and potential for, quantum cutting layers to be integrated into tandem cells has not yet been assessed. As such, we used detailed balance analysis to investigate the impact of Yb:CsPb­(Cl_1–*x*_Br_*x*_)_3_ quantum cutting materials for idealized single junction and perovskite/silicon tandem solar cells. Figure 1a schematically summarizes the approach and assumptions used in these calculations. Above the QC_gap_, the sunlight is completely absorbed by the quantum cutting layer and converted to NIR photons with a PLQY of 200% (the theoretical limit). Each solar cell is also assumed to have a step function absorption spectrum at its respective band gap with complete sub-bandgap transmission. In the interest of finding the thermodynamic limit to PCE (PCE_max_), we have assumed that all photons emitted by the quantum cutting layer are emitted in the direction of the underlying solar cell(s). This quantum cutting layer substantially modifies the AM1.5G spectrum incident on the underlying solar cell (Figure 1b). With decreasing QC_gap_ (from yellow to purple, Figure 1b), the high-energy light is eliminated at progressively lower energies and a large peak in the NIR, centered at 1.25 eV, grows due to the quantum cutting emission. We have followed the procedure laid out by Kirchartz et al. to conduct the detailed balance analysis (see Supporting Information for more details) for varying values of QC_gap_.^37^ Since the threshold for quantum cutting in Yb:CsPb­(Cl_1–x_Br_*x*_)_3_ is 2.53 eV, we have restricted the calculations to a QC_gap_ **≥** 2.53 eV.^33^ Although we focus on the use of Yb:CsPb­(Cl_1–x_Br_*x*_)_3_, we expect the results here to be broadly generalizable to any quantum cutter relying on NIR emission from an Yb^3+^ dopant, though there may be subtle differences for emitters with different PL profiles.

**Figure 1:**
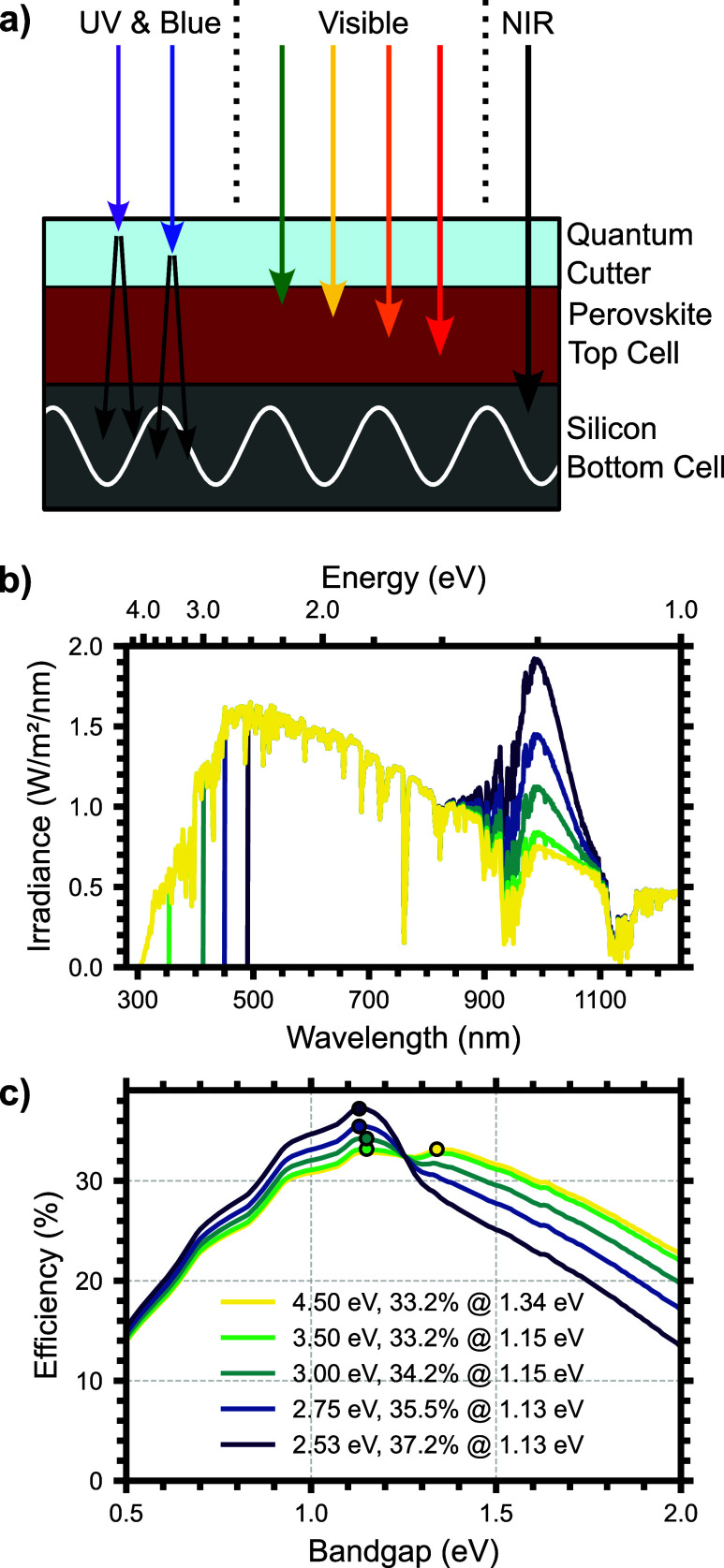
Schematic of quantum cutting above the quantum cutting bandgap (a). AM1.5G irradiance incident on a solar cell after modification by quantum cutting (b) and the resulting efficiency limit for a single junction solar cell (c) plotted as a function of quantum cutting bandgap from no quantum cutting (yellow) to the quantum cutting threshold (purple). The maximum PCE and the energy are noted in the legend and labeled on the plot as a data point.

Quantum cutting increases the detailed balance limit for a single junction solar cell with a bandgap below the quantum cutting emission (Figure 1c). We plot the single junction efficiency limit as a function of the single junction bandgap (E_g,SJ_) for varying values of the QC_gap_ ranging from no quantum cutting layer (yellow) to a QC_gap_ of 2.53 eV (purple) with the PCE_max_ point labeled. With a progressively smaller QC_gap_, a larger number of high-energy photons are converted to the NIR with photon multiplication, resulting in an increase in the maximum efficiency for a solar cell with a bandgap below the quantum cutting emission energy. On the other hand, for values of E_g,SJ_ above the quantum cutting emission energy, the efficiency is reduced due to the reduced photocurrent. As a result of the change in spectral shape, the PCE_max_ shifts from 33.2% at E_g,SJ_ = 1.34 eV to 37.2% at E_g,SJ_ = 1.13 eV, an increase of 4 absolute % points. In particular, the quantum cutting emission is well-suited for the bandgap of silicon (1.12 eV),^32^ increasing its PCE_max_ from 32.7% to 37.2%, an increase of 4.5 absolute % PCE, confirming that photon multiplication by quantum cutting improves the maximum theoretical efficiency. Despite this improvement, quantum cutting as a strategy for improving single junction solar cell efficiency does not approach the theoretical 45% PCE_max_ of a two-junction tandem solar cell,^37–39^ and is only slightly higher than the demonstrated efficiency of perovskite-on-silicon tandem solar cell, at 35%.^40^

We now apply the same detailed balance analysis method to a two-junction tandem solar cell with a quantum cutting layer modifying the AM1.5G solar spectrum. We show the PCE contour plots for arbitrary bottom and top cell bandgaps in Figure 2 for three quantum cutting configurations: no quantum cutting layer (Figure 2a,d), a pure Yb:CsPbCl_3_ quantum cutting layer (QC_gap_ = 3.06 eV, Figure 2b, 2e), and the mixed halide Yb:CsPb­(Cl_0.25_Br_0.75_)_3_ with the QC_gap_ at the quantum cutting threshold (QC_gap_ = 2.53 eV, Figure 2c, 2f). The PCE_max_ is marked with a red point for each QC_gap_ and tandem architecture with the optimal top and bottom cell bandgaps noted. Surprisingly, despite the photon multiplication, the drop in voltage of the tandem solar cell with quantum cutting integration almost entirely balances the increase in net current density, and the PCE_max_ remains largely unaltered, with a marginal reduction for the two-terminal (2T) tandems, and a marginal increase for the four-terminal (4T) tandems. However, the optimum band gaps for the two junctions are significantly influenced. Current matching in the 2T architecture forces the top bandgap to shift to substantially lower energy. The quantum cutter not only increases photon flux to the bottom cell but also reduces the photon flux to the top cell by absorbing the high-energy light. As a result of these effects, the optimal combination of bandgaps shifts from 1.63 eV/0.96 eV (top/bottom) to 1.38 eV/0.95 eV. As expected, the optimal combination of bandgaps is less affected for the 4T architecture since it is not limited by current matching, but instead, for low values of QC_gap_, the optimal bottom cell shifts to higher energy. In this case, the reduced thermalization of the NIR photons due to a larger band gap, particularly those emitted by the quantum cutter, outweighs the increased photocurrent available for a bottom cell with a bandgap of 0.94 eV. Silicon, with a bandgap of 1.12 eV, becomes the ideal bottom cell for a 4T tandem with quantum cutting. Another region of interest for 4T tandems with quantum cutting is the 42–44% efficient near-optimal plateau with a top cell bandgap of ∼1.1 to 1.3 eV and a bottom cell bandgap of ∼0.6 to 0.9 eV. This combination of bandgaps is not typically efficient enough to warrant further investigation but may be an opportunity for tandem solar cells with a silicon or low bandgap perovskite top cell and a germanium bottom cell. The PCE_max_ changes negligibly for both 2T and 4T architectures when adding a QC layer, decreasing from 44.9% to 44.2% for the 2T architecture, and increasing slightly from 45.3 to 45.4% for the 4T architecture with a QC_gap_ close to the quantum cutting threshold for Yb^3+^. Notably, the quantum cutting emission is too low in energy to be a viable match with an all-perovskite tandem in which the bottom Pb–Sn alloyed perovskite often has a bandgap ≥ 1.24 eV.^41^ The corresponding plots together with a short discussion for the AM0 spectrum, which contains substantially more UV light, can be found in the Supporting Information.

Surprisingly, despite the photon multiplication, the drop in voltage of the tandem solar cell with quantum cutting integration almost entirely balances the increase net current density, and the PCE_max_ remains largely unaltered.

**Figure 2:**
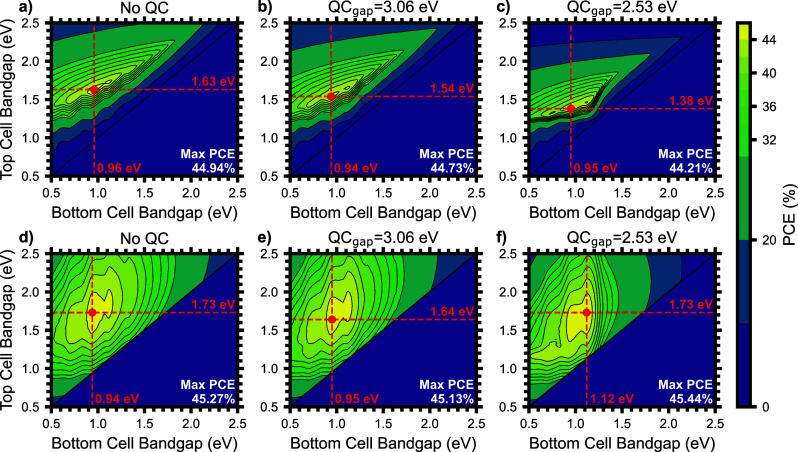
Contour plots of the power conversion efficiency as a function of the bottom (*x*-axis) and top cell (*y*-axis) bandgaps for a two-junction tandem solar cell in two-terminal (a–c) or four-terminal (d–f) configurations. Plots are varied for no quantum cutting layer (a, d), Yb:CsPbCl_3_ (b, e), or mixed Yb:CsPb­(Cl_*x*_Br_1–*x*_)_3_ with quantum cutting bandgap of 2.53 eV (c, f). The maximum power conversion efficiency is noted in the lower right of each plot, and the combination of top and bottom cell bandgaps is noted in red.

As previously discussed in the case of the single junction results, the quantum cutting emission is a good match for the bandgap of silicon. The silicon bottom cell bandgap (1.12 eV) becomes the near-ideal bottom cell after spectral shaping by quantum cutting. To investigate this further, we have plotted the PCE limit of silicon-based 2T and 4T tandem cells with a varying QC_gap_ on the *y*-axis and varying top cell bandgap on the *x*-axis (Figure 3). The current matching requirements of the 2T configuration are evident in the relatively narrow optimal PCE maximum (Figure 3a). The optimal top cell bandgap for a 2T tandem decreases significantly from 1.73 to 1.46 eV with decreasing QC_gap_ from 4.5 to 2.53 eV. This result can be understood by the current matching constraints of the 2T tandem configuration. With a reduced QC_gap_, fewer photons are absorbed by the top cell, producing less current in the top cell, even as the photon multiplication of the quantum cutting layer increases the current produced in the bottom cell at twice the rate of current reduction in the top cell. To continue matching the current produced by the bottom cell, the top cell bandgap must decrease, leading to a 290 meV decrease in the optimum top cell bandgap. Since the top cell bandgap decreases to accommodate the change in currents from the top and bottom cells, the photovoltage per photon also decreases, leading to a nearly constant PCE_max_ for the 2T tandem ranging from 44.4 to 44.1% PCE. On the other hand, a similar dynamic is evident in the 4T tandem but is significantly less pronounced since there is no need to match currents between the top and bottom cells. In this case, the optimal top cell bandgap decreases only slightly to retain a correspondingly high photovoltage from these higher energy absorbed photons. In the 4T configuration, the PCE_max_ increases slightly by 0.8 absolute % from 44.6% to 45.4% PCE.

The optimal top cell bandgap for a 2T tandem decreases significantly from 1.73 to 1.46 eV with decreasing QC_gap_ from 4.5 to 2.53 eV.

**Figure 3:**
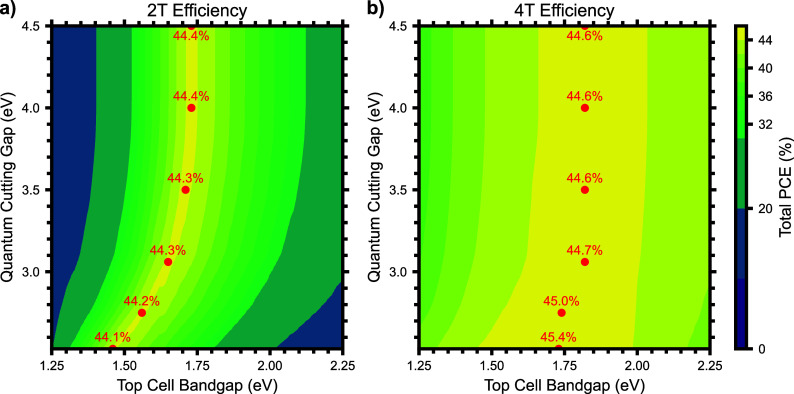
Power conversion efficiency for perovskite/silicon tandem solar cells with varying top cell bandgaps (*x*-axis) and quantum cutting bandgaps (*y*-axis) for the 2-terminal (a) and 4-terminal (b) architectures. The maximum power conversion efficiency for various quantum cutting bandgaps is noted in red with a point at the optimal top cell bandgap.

While we have modeled the ideal case, we expect similar trends to occur even in practical, nonideal devices. Broadly, there are two significant possibilities for energy losses from the quantum cutting layer: (1) less than 100% absorption by the quantum cutting layer and (2) less than 200% quantum efficiency of photons above the quantum cutting bandgap. In the case of incomplete absorption by the quantum cutting layer, since photocurrent must be matched by the top and bottom cells, the ideal top cell bandgap will not decrease as significantly. This is a result of additional absorption of high energy photons by the top cell and hence, fewer photons that the quantum cutter is absorbing and multiplying for the bottom cell to harvest. However, since the photon multiplication of quantum cutting does not have a substantial effect on the tandem efficiency, reduced absorption by the quantum cutting layer would also not substantially change the overall efficiency. Less than 200% external quantum efficiency above the quantum cutting bandgap could be the result of less than 200% emission by the quantum cutting layer and/or less than 100% absorption efficiency of the emitted photons by the solar cell. In this case, the trend to a lower ideal top cell bandgap will similarly be reduced in magnitude as the bottom cell photocurrent will be reduced from the ideal quantum cutting scenario modeled here. Any decrease in the overall emission or recollection efficiencies will impact the maximum attainable PCE since this power is lost rather than transmitted to the underlying cell. As an example, we calculated the efficiency limit for a perovskite/silicon tandem with a reduced external quantum efficiency above the quantum cutting gap (150% instead of the ideal 200%), which is plotted in Figure S5 in the Supporting Information. The same overall trend is visible, but the maximum efficiency decreases to 42.3% (2T) or 43.2% (4T) with quantum cutting. The optimal top cell bandgap still shifts to lower energy as compared to the cell without a quantum cutter, but to a slightly lesser extent (1.50 eV for 2T and 1.71 eV for 4T).

## Shift in Ideal Top Cell Bandgap

There is evidentially no substantial efficiency gain from integrating QC materials into the idealized tandem solar cells which we have modeled. However, reducing the optimal top bandgap of a perovskite/silicon tandem may present a significant advantage and an opportunity for spectral shaping by quantum cutting. Under AM1.5G, the optimal 1.68–1.74 eV bandgap is typically achieved by using a mixed-cation, mixed-halide perovskite. These perovskites can suffer from a variety of instabilities, particularly halide segregation.^12^ Using a quantum cutting layer with a QC_gap_ = 2.53 eV would enable the use of a neat iodide top cell, such as FAPbI_3_ (bandgap of 1.48–1.53 eV)^42,43^ without significant losses from current matching. Without quantum cutting, a 2T FAPbI_3_/silicon tandem solar cell would have a detailed balance limit of 26.6–30.4%, assuming a step function absorption onset from zero to complete absorption at 1.48–1.53 eV (respectively) for the FAPbI_3_. We do note that in a practical realization of a tandem cell using FAPbI_3_ and Si, the thickness of the perovskite absorber could be reduced to match the current of the bottom silicon cell, leading to a thermodynamic efficiency limit of 39.3–40.2% under AM1.5G illumination (see Figures S6 and S7 for further details using a thinned top cell). This is still significantly lower than the optimal ∼45% 2T tandem limit but greater than the single junction silicon 32.7% PCE_max_. With the ideal quantum cutting layer, the efficiency limit of a 2T FAPbI_3_/silicon tandem is 44.1–44.2%, an increase of 4.0–4.8 absolute % PCE in comparison with the 2T limit employing an optically thinned perovskite layer. The stability of neat iodide perovskites or perovskites with minimal bromide can be over an order of magnitude higher.^44^ This indicates that incorporating a quantum cutting layer and employing a neat iodide perovskite top cell could improve the stability of perovskite/silicon tandems significantly without sacrificing PCE.

Reducing the optimal top bandgap of a perovskite/silicon tandem may present a significant advantage and an opportunity for spectral shaping by quantum cutting.

These lower bandgap perovskite cells are also superior to the wider bandgap cells from a practically realized efficiency standpoint. An analysis of the external radiative efficiencies of conventional top cells for perovskite/silicon tandems indicates that they have lagged behind those of lower bandgap single junction cells.^11^ By shifting the solar spectrum to lower energies, the perovskite/silicon tandem solar cell community can adopt some of the best-performing single junction perovskite solar cell absorbers and device contacts, making significant strides in tandem efficiency without needing to further develop ∼1.7 eV top cells. This is particularly critical to achieve both high efficiency and stability since many strategies for passivating ∼1.7 eV top cells rely on primary amines, a known source of instability in operation under light and heat.^45^

## Improved External Quantum Efficiency

Spectral shaping via quantum cutting can also have practical benefits for improving the PCE of silicon/perovskite tandems because of differences in external quantum efficiency (EQE) for photocurrent generation between the UV and NIR.^15^ Crane et al. reported that several silicon single junction technologies exhibit poor EQE above 2.5 eV, improving the PCE achievable in these single junction technologies by 3.5–5.3 absolute % PCE.^7^ This range was dependent on the EQE of the silicon in both the high-energy spectral region, where the quantum cutting layer absorbs, and at the wavelengths the quantum cutting layer emits. Perovskite/silicon tandem solar cells also typically have a worse EQE in the UV, often due to parasitic absorption by the top contact, but it is typically not as bad as the EQE of single junction silicon in this region.^7,46^ However, quantum cutting can improve the energy yield even for the current record perovskite/silicon tandem solar cell.^40^ After digitizing the published EQE for the perovskite and silicon subcells, we calculated the power generated by the high-energy spectral region and what could be generated by the quantum cutting emission. The high-energy spectral region of the perovskite EQE was multiplied by the AM1.5G spectrum and weighted by the maximum power point voltage of the perovskite, resulting in 53.7 W/m^2^ generated by the top cell. Converting these photons to NIR via quantum cutting, multiplying by the silicon subcell EQE, and weighting that by the silicon maximum power point voltage gives 63.5 W/m^2^. From this analysis, we conclude that the addition of a quantum cutting layer could improve the overall power output by 9.9 W/m^2^, or about 1% absolute PCE. Critically, this simple analysis does not take into account the current matching of these two subcells in 2T architecture and hence does not consider the slightly lower optimal bandgap for the top cell and corresponding voltage drop. However, in realistic 4T tandem architecture this power gain of 9.8 W/m^2^, about 1 absolute % PCE, may be realizable.

## UV Filtering

Lastly, the addition of a quantum cutting layer could have significant benefits for UV stability, a challenge for single junction silicon and perovskite/silicon tandem solar cells alike.^13,47–49^ The quantum cutting layer effectively acts as a UV filter, a strategy that has been employed by the solar industry in the past in cerium doped glass.^50,51^ Instead of losing this energy, however, using a quantum cutting layer as the UV filter could enable the silicon cell to harvest this energy. This could not only improve the UV stability of the laminate, but also the underlying perovskite/silicon tandem device stack. In fact, the stability-enhancing effect of the quantum cutting layer has already been observed for “integrated perovskite-organic” solar cells that employ both perovskite and organic light absorbers without intermediate charge transport or tunnel junction layers.^52–54^ Here, the devices containing a quantum cutting layer retained 90% of their initial efficiency after 1000 h of UV illumination, while the control sample fell below that threshold after about 500 h.^52,53^ Nevertheless, relying on the quantum cutting layer to prevent UV-induced degradation requires further development of UV stress testing standards and understanding how the quantum cutting material itself degrades with light and heat.^55^ For example, the perovskite host lattice could undergo photochemical degradation under UV irradiation, which could shift the bandgap and degrade the material, gradually reducing the UV absorption efficiency and NIR emission.^56^

The addition of a quantum cutting layer could have significant benefits for UV stability, a challenge for single junction silicon and perovskite/silicon tandem solar cells alike.

The relative benefit of using a quantum cutting layer for UV protection is expected to be strongly dependent on the threshold of damage to the underlying device as well as the UV stability and performance of the quantum cutting layer. For example, cerium-doped glass with a transmission of 50% at ∼350 nm would reduce the ideal perovskite subcell photocurrent by ∼3%,^50^ whereas using a 435 nm cutoff filter^13^ would represent a ∼12% decrease in the ideal perovskite subcell photocurrent. Since the energy threshold and magnitude for UV damage in perovskite/silicon tandems is not well established and may vary significantly depending on the device architecture, the relative cost benefit analysis of using a standard industrial filter versus a quantum cutting layer will be strongly dependent on both the specific device architecture and performance.

## Performance in Single Junction Solar Cells

The addition of a quantum cutter to a tandem solar cell has not yet been reported, but quantum cutters have been reported in single junction solar cells. The majority of these studies have focused on glasses or ceramics as quantum cutting materials, as reviewed by Chen et al.,^57^ but relatively few solar cells benefiting from the incorporation of rare-earth doped halide perovskites have been reported. We have summarized these reports in Table 1 with the relative increase in PCE attributed to the addition of the quantum cutting layer. Notably, these reports all come from one research group, indicating the strategy has not been successfully adopted by a broad range of researchers. The results vary strongly, with relative increases in PCE attributed to quantum cutting ranging from 3.9%^58^ to 20.1%.^5,59^ It is difficult to deconvolute the effect of quantum cutting itself versus other optical effects, particularly an antireflective (AR) effect of the quantum cutting layer. Some publications take care to deconvolute this,^5,26,60^ but it is also frequently overlooked, leading to an overestimation of the impact of quantum cutting on PCE. So far, a quantum cutting layer has been tested for silicon, copper indium gallium selenide,^5,59,61–63^ and integrated perovskite-organic^52,53^ solar cells. The data in Table 1 reveal that the addition of a quantum cutting layer has its greatest effect on solar cells with a suitably low bandgap in which the quantum cutting emission is well matched with the solar cell EQE. In contrast, the relatively higher bandgap of the integrated perovskite-organic solar cell only sees a modest improvement with the addition of a quantum cutting layer. Even this improvement is surprising given the low absorbance of the quantum cutter and poor match of the EQE with the quantum cutting emission.

**Table 1:** Overview of Solar Cells with Increased PCE due to a QC Layer^a^

Year	QC material	Solar cell type	PCE(control) (%)	PCE(with QC) (%)	Relative increase in PCE (%)	Notes	Ref
2017	Ce^3+^,Yb^3+^:CsPbCl_1.5_Br_1.5_ NCs	c-Si	18.1	21.5	18.8		^5^
2019	Yb^3+,^Pr^3+^,Ce^3+^:CsPbClBr_2_ NCs	CIGS	15.9	19.1	20.1	PCE was also increased for other QC materials with PLQY < 100%, indicating the increase cannot be attributed to QC alone	^59^
2022	Yb^3+^,Pr^3+^,Cr^3+^:CsPbCl_3_ NCs in PMMA matrix	PVK/BHJ	21.97	23.40	6.5	focus on optimizing BHJ, effect of undoped CsPbCl_3_ as reference not reported	^52^
2024	Yb^3+,^Li^+^:CsPbCl_3_ NCs	PVK/BHJ	22.85 (with optimized BHJ)	23.7524.21 (with add. MgF_2_ AR coating)	3.9	AR effect of QC layer not evaluated	^53^
2024	Yb^3+^:CsPbCl_3_ bulk film (passivated with chlorophyll)	c-Si	21.41	23.22	8.4	AR effect of QC layer not evaluated	^61^
2024	Yb^3+,^Ru^3+^:CsPbCl_3_ NCs (passivated with cysteine)	c-Si	21.45	23.15	7.9	AR effect of QC layer not evaluated	^62^
2024	Yb^3+,^Zn^2+^:CsPbCl_3_ NCs	c-Si	18.6	21.2	14.0	AR effect of QC layer not evaluated; only champion device reported	^63^

a Solar cell type: c-Si = monocrystalline silicon; CIGS = CuIn_*x*_Ga_1–*x*_Se_2_; PVK/BHJ = integrated perovskite-organic solar cell. If available, the average PCE is reported instead of the champion result.

In this case, the absorbance of the quantum cutting layer was only about 30–50%, so relatively few photons benefited from photon multiplication. Additionally, the EQE in the NIR region of the quantum cutting emission ranges from 2 to 29% (based on the PL full width half max), indicating poor response of the solar cell to NIR light emitted by the quantum cutter. Lastly, the solar cell EQE in the UV region of the quantum cutter absorption ranges from 1.4 to 77%, indicating that this perovskite with bulk organic heterojunction has reasonable charge carrier conversion in this region. Furthermore, the results in this perovskite with organic heterojunction solar cell are inconsistent with other literature on the working principle of these types of cells, indicating that significant questions remain on the efficacy of this strategy.^54^

The addition of a quantum cutter to a tandem solar cell requires solving several problems: (1) the method of incorporating these materials into a solar cell stack, (2) the synthesis and morphology of the quantum cutting material, and (3) the stability of a high PLQY material over the course of more than 25 years.

## Incorporation of Quantum Cutting Layer

The strategy for incorporating the quantum cutter in the solar cell device stack could dictate both the real-world performance and stability of the quantum cutter. This has largely been unaddressed by the reports in Table 1. In most of these reports, the quantum cutting layer was deposited directly on the solar cell and was unencapsulated^5,59,61,63^ or the quantum cutting layer was deposited on top of the glass.^52,53^ Only one report incorporated the quantum cutting layer into a fully encapsulated device, but this report did not include details on the encapsulation process, which may or may not be industrially compatible.^62^ Since the perovskite is unlikely to survive direct exposure to water, these strategies are insufficient, and the optimal method of incorporation has not been solved. Optical modeling by Keil et al. revealed differences in light coupling depending on how the quantum cutting material is incorporated for single junction solar cells.^6^ The addition of a film to the underside of the cover glass had the worst performance, with an increase in reflection and trapped modes, resulting in less than 30% of light coupling. Deposition on textured solar glass, as opposed to flat cover glass, may reduce reflection and light trapping of light emitted by the quantum cutting layer. However, light coupling can be increased to over 80% by either depositing the quantum cutting layer within the texture of a single junction silicon solar cell or incorporated into an encapsulant to form a nanocomposite.

Both optimal strategies for incorporating a quantum cutter could be applied to a 2T tandem but could be challenging for a 4T architecture because of the relatively flat nature of transparent conducting oxide (TCO) coated glass. The performance of a quantum cutter deposited directly onto a textured perovskite/silicon tandem cell would likely be strongly dependent on the texture and conformality of the quantum cutter since these affect reflection and light trapping. Nevertheless, deposition on a highly textured silicon substrate has been demonstrated on a lab scale, indicating it could be a viable strategy.^3^ Similarly, while so far unproven, incorporation of a quantum cutter into the encapsulant as a nanocomposite is a straightforward method to incorporate the quantum cutter for a 2T tandem cell. In a 4T architecture, on the other hand, the perovskite submodule is typically deposited directly on the cover glass and operated in superstrate configuration to keep glass use and costs at a minimum. This architecture precludes the ability to add a quantum cutting layer before the perovskite submodule absorbs the UV and visible light. A 4T tandem with quantum cutting could be created by precoating the glass substrate with the quantum cutting layer, prior to TCO deposition, but this may rule out use of the most ubiquitous thin-film TCO, fluorine doped tin oxide, due to the high temperature of deposition. Additionally, reflectance at the flat interfaces of the quantum cutter may lead to significant reflectance losses. Alternatively, a 4T tandem could incorporate a nanocomposite quantum cutting material as the encapsulant between a sheet of cover glass and the perovskite submodule substrate, but this would increase glass usage and cost and would likely still result in optical waveguiding within the front sheet of the PV module glass and optical loss out the sides of the modules.

## Synthesis of Quantum Cutting Material

The method for incorporating the quantum cutting layer could also restrict the synthetic method used to synthesize Yb:CsPb­(Cl_1–*x*_Br_*x*_)_3_. Most publications focus on nanocrystals formed by hot injection of either a cesium^4,35,64^ or halide^1,34^ source into a lead precursor solution. While nanocrystals would be straightforward to incorporate into a nanocomposite with an encapsulant for high optical coupling, their synthesis and ligand exchange present several challenges for scalable manufacturing. It is also possible to prepare Yb:CsPb­(Cl_1–*x*_Br_*x*_)_3_ as a bulk thin-film using a two-step spin coating process or vapor deposition. For solution coating, the substrate is first coated with a solution of the lead halide, then the cesium halide, before an annealing step to form the perovskite. The ytterbium halide can be included in either the lead or cesium precursor solution.^2,36,61^ For vapor deposition, a single precursor is first prepared simply by ball milling the metal halides, including the dopant, before flash evaporating it onto the substrate.^3,65^ Solvent compatibility limits direct solution deposition on a 2T tandem solar cell since the solvents may penetrate the device stack and dissolve the underlying perovskite subcell. Additionally, the 250 °C annealing required to form the Yb:CsPb­(Cl_1–*x*_Br_*x*_)_3_ is a harsh temperature for the underlying perovskite/silicon tandem solar cell.^2^ Vapor deposition could deposit the quantum cutting layer without these challenges and has already been shown to deposit conformally on textured silicon solar cells, but the solar cell performance has not been reported for either single or double junction devices.^3^ For all three methods, nanocrystals, solution-processed, and evaporated films, PLQY values of above 180% have been reported, promisingly close to the theoretical maximum of 200% that has been the basis of our detailed balance calculations.

## Photothermal Stability and Performance

Regardless of how the quantum cutting layer is added to the solar cell device stack, these materials must demonstrate exceptional stability to be a serious contender for spectral shaping. At the front of the device stack, the quantum cutter is exposed to the unfiltered solar irradiation (particularly the UV) and must be thermally stable. While UV stability has received some attention in the broader perovskite community and specifically for the Yb:CsPb­(Cl_1–*x*_Br_*x*_)_3_ composition, this has not received the level of scrutiny it needs. For example, the UV stability of Yb:CsPb­(Cl_1–*x*_Br_*x*_)_3_ nanocrystals was tested by Jing et al. at approximately 1-sun UV fluence (but under monochromatic 365 nm light).^4^ The PL of C3-sulfobetaine-ligated nanocrystals decreased to 80% of their initial value over the course of approximately 2500 h, but seemed to be more stable after this time up to 6000 h. The authors, however, do not report the initial PLQY of these samples or whether this stability is measured in the solution or solid-state. Nevertheless, this study highlights the importance of ligation for high PLQY in quantum cutting materials, since the PL of nanocrystals with typical oleylammonium and oleate ligands decreased to 20% of their initial value. The quantum cutting layer must also exhibit exceptional stability to high temperature. This was tested for 1000 h at 80 °C under an argon atmosphere using normalized PL, revealing a drop of 14% for the C3-sulfobetaine-ligated nanocrystals compared to a drop of 60% for the oleylammonium and oleate ligated nanocrystals. Given that these stability data use relative PL intensity rather than PLQY, this gap raises the possibility that these more stable materials are not necessarily champion performers. Additionally, while these data focus on nanocrystals, the material stability for solution-processed or evaporated bulk thin films could be substantially different and requires further investigation. To merit inclusion in a commercial product, quantum cutters must reach significant stability milestones under both light and heat stress, similar to the stress tests used for solar cells.^55^

A further major obstacle to the utility of quantum cutting Yb:CsPb­(Cl_1–*x*_Br_*x*_)_3_ is their stability and performance under high excitation fluence. Kroupa et al. found that the PLQY of the quantum cutter significantly decreases under high excitation fluence.^2^ A mixed halide Yb:CsPb­(Cl_1–*x*_Br_*x*_)_3_ thin-film with 190% PLQY at low excitation fluence had approximately 30% PLQY under higher intensity excitation (10^16^ photons/(cm^2·^ s)), but this excitation intensity is still less than one third of the AM1.5G flux above the Yb:CsPb­(Cl_1–*x*_Br_*x*_)_3_ bandgap. Erickson et al. used two-pulse PL experiments to identify that further excitation of perovskite containing excited Yb^3+^ dopants resulted in saturation of the NIR emission.^66^ They hypothesized that a second excitation event results in a rapid nonradiative Auger-type relaxation process, reducing the overall PLQY and energy yield, which could be mediated by halide-to-ytterbium charge transfer states. These data suggest that the NIR PL saturation is an intrinsic material limitation due to the long millisecond lifetime of the excited Yb^3+^ state. Despite this limitation, there are opportunities for reducing its impact under 1-sun fluences through careful material and/or device design. For example, increasing the Yb^3+^ concentration reduced the photoexcitation rate per Yb^3+^ and decreased the PL saturation.^66^ Curiously, nanocrystal samples with a similar level of doping but using a zwitterionic C3-sulfobetaine ligand demonstrated significantly reduced the PL saturation.^4^ Nanocrystalline quantum cutting samples across three publications^1,4,66^ are reported to have reduced PL saturation as compared to their bulk counterparts.^2^ This may be the result of the smaller likelihood that a nanocrystal with an excited Yb^3+^ dopant absorbs a second high energy photon or that electronic transport within bulk Yb:CsPb­(Cl_1–*x*_Br_*x*_)_3_ films plays a significant role in nonradiative relaxation between excited charge carriers at the semiconductor band edge and an excited Yb^3+^ dopant. One critical experiment that we have not seen reported is the reversibility of this PL saturation. To eliminate the possibility of degradation during the measurements, the samples could be measured at incrementally higher fluences until the maximum fluence, then measured again with incrementally lower fluences. If the PL saturation is merely a mechanistic result of the long Yb^3+^ lifetimes, the PLQY at each lower excitation fluence should be reproducible after saturation is reached at high excitation fluences. On the other hand, the apparent observation of PL saturation could be the effect of light-induced degradation of the quantum cutting layer, requiring an improvement in stability. Erickson et al. used a random time delay in their two pulse PL experiments, which reduces the likelihood that these experiments are actually probing photoinduced degradation, but is not as conclusive as repeated measurement at low and high fluences.^66^

As a final consideration, a photovoltaic module will rarely operate at room temperature and may be at temperatures ranging from 35 to 85 °C during sunny days, depending upon geographic location and season. Band-to-band radiative recombination diminishes considerably for metal halide perovskites as the temperature is increased. The quantum cutting efficiency has been studied as a function of temperature below room temperature, particularly to cryogenic temperatures to better understand the mechanism.^67^ The mechanistic insights from this work suggest that quantum cutting should be the dominant emission mechanism at elevated temperatures due to strong overlap between the perovskite band edge donor and Yb^3+^–Yb^3+^ dopant acceptor electronic transitions. The quantum cutting emission strength in Yb:CsPbCl_3_ (having a bandgap much greater than the quantum cutting threshold) was found to have no correlation with temperature, while alloyed Yb:CsPb­(Cl_1–*x*_Br_*x*_)_3_ (bandgap near the quantum cutting threshold) demonstrated significant quantum cutting quenching at reduced temperature. These results indicate that the quantum cutting mechanism in Yb:CsPb­(Cl_1–*x*_Br_*x*_)_3_ should not limit the quantum cutting efficiency at device-relevant temperatures, which is consistent with data on Yb and Er co-doped CsPbCl_3_ nanocrystals.^68^ However, as far as we are aware, there are no reports on how the quantum cutting efficiency in Yb:CsPb­(Cl_1–*x*_Br_*x*_)_3_ varies at elevated operational temperatures, which must be confirmed for the effective integration of these materials as quantum cutters into a useful photovoltaic module. The stability of nanocrystal samples has been tracked while annealing at 80 °C, demonstrating that these quantum cutters retained 86% of their initial PL after 1000 h of continuous annealing.^4^ This report only measured relative PL intensity rather than absolute PLQY during or after heating, so is not indicative of the performance of quantum cutters operating under high heat. This is clearly a consideration which needs to be addressed.

Further understanding of the temperature, spectral, and fluence dependence of quantum cutting will also be crucial to understanding the overall energy yield of tandems that incorporate quantum cutting layers. Modeling of single junction silicon cells paired with quantum cutters revealed a potential increase in power output, but simultaneously revealed sensitivities to fluence over the course of different times of the day or year as the solar spectrum changes.^7^ These sensitivities, coupled with the current limitations of 2T tandem cells, raises questions of energy yield as the incident solar spectrum shifts. Energy yield calculations for 2T versus 4T tandems show a sensitivity to the average photon energy.^69^ At times when the average photon energy is greater than the average AM1.5G photon energy (i.e., when the bottom cell is current limiting), a quantum cutting layer could increase the energy yield by shifting more photons to the bottom cell. On the other hand, at times when the average photon energy is less than the AM1.5G average (i.e., when the top cell is current limiting), the quantum cutting layer would exacerbate the spectral imbalance, reducing the overall energy yield. Understanding the practical effects for different locations and times of day and year will require more careful understanding of device performance of a fabricated quantum cutter/perovskite/silicon tandem, including understanding spectral and fluence dependence of performance.

## Quantum Cutting Mechanism

Further investigations of the quantum cutting mechanism could shed light on these performance limitations and help identify strategies for synthesizing the ideal quantum cutting layer. Until recently, quantum cutting was identified by changes in the time-resolved PL or macroscopic PLQY. Photon correlation analysis has been used to identify quantum cutting in yttrium phosphate co-doped with Tb^3+^ and Yb^3+^ and disprove quantum cutting in Ce^3+^ and Yb^3+^ co-doped yttrium aluminum garnet, in which photons are merely down converted without multiplication.^70^ Given the significant technical challenges of measuring absolute PLQYs accurately,^71^ these photon correlation experiments would be a valuable addition to the understanding of quantum cutting in Yb:CsPb­(Cl_1–*x*_Br_*x*_)_3_. These could also be coupled with structural characterization since doping induced defects have been hypothesized to act as intermediary states for energy transfer.^1,2^ Since the ytterbium ion has a +3 charge and there is no 3+ cation in the perovskite host lattice, incorporation of the ytterbium must induce a defect to compensate for this charge imbalance. Multiple groups have investigated the problem using density functional theory calculations,^64,72,73^ high-resolution optical spectroscopy,^74,75^ and atomic resolution scanning tunneling electron microscopy and energy dispersive spectroscopy imaging,^76^ and found that the Yb^3+^ ions mostly occupy Pb^2+^, rather than Cs^+^ or interstitial sites, and that the charge is most likely compensated by the formation of one lead vacancy (V_Pb_) for every two dopant ions. Simulations suggest that the two Yb^3+^ dopants and V_Pb_ form clusters with a preference for a right-angled Yb^3+^–V_Pb_–Yb^3+^ complex, though the formation energies for linear or random configurations are only marginally higher.^73^ The mechanism has been investigated indirectly by studying nanocrystals with isoelectronic gadolinium co-dopants and in Yb:CsPbCl_3_ single crystals, suggesting that the quantum cutting mechanism is robust to the local environment of the Yb.^74,75^ Further investigations could help explain the differences leading to quantum cutting in some materials but down conversion in others.

In conclusion, Yb:CsPb­(Cl_1–x_Br_*x*_)_3_ perovskite quantum cutters could create a host of new opportunities for perovskite/silicon tandem solar cells. By shifting the solar spectrum from the UV and high energy visible to the NIR, the optimal top bandgap is shifted to lower energies. Perovskite solar cells with these lower bandgaps overcome certain instabilities in the perovskite absorber layers and have already yielded device efficiencies closer to their thermodynamic efficiency limit than their wider gap counterparts. This spectral shaping allows for the use of these potentially superior materials without substantial efficiency losses. To achieve this promise, however, quantum cutters must be synthesized and incorporated into a device with high PLQY under solar irradiances and operating temperatures, excellent optical coupling, and long-lasting stability under light and heat.

## Supplementary Material



## Notes

Description of detail balance analysis method; results of detail balance analysis for solar cells under AM0 illumination; power conversion efficiency limit of perovskite/silicon tandem cells with reduced quantum efficiency and with top cell thinning (PDF)
