# Supplementary material for: Assessing the Opportunities of Spectral Shaping by Quantum Cutting for Perovskite/Silicon Tandem Solar Cells
Source: ACS Energy Lett. 2026 Apr 17;11(5):3664–75. doi: 10.1021/acsenergylett.6c00270 (PMC13170752; doi:10.1021/acsenergylett.6c00270)
Supplement: Supplementary file 1 [file nz6c00270_si_001.pdf]

Supporting Information for

Assessing the Opportunities of Spectral Shaping by Quantum Cutting for Perovskite/Silicon Tandem Solar Cells

Brian M. Wieliczka,[1,] Jakob Möbs,[1-3] Nakita K. Noel,[1] Henry J. Snaith[1,]**

[1] Clarendon Laboratory, Department of Physics, University of Oxford, Oxford, United Kingdom, OX1 3PU, [2] Institute for Inorganic and Analytical Chemistry, Justus-Liebig-University Gießen, D-35392 Gießen, Germany, [3] Center for Materials Research (LAMA), Justus-Liebig-University Gießen, Heinrich-Buff-Ring 16, 35392 Gießen, Germany

Corresponding Authors:

*brian.wieliczka@physics.ox.ac.uk

*henry.snaith@physics.ox.ac.uk

TABLE OF CONTENTS

Detailed Balance Analysis Method S2

Modified Spectrum after Quantum Cutter under AM0 Illumination S6

Efficiency Limit of Single Junction Solar Cell with Quantum Cutting under AM0 Illumination S7

Power Conversion Efficiency of Tandem Solar Cells with Arbitrary Band Gaps under AM0 Illumination S8

Power Conversion Efficiency of Perovskite/Silicon Tandem Solar Cells with Quantum Cutting Layers under AM0 Illumination S9

Power Conversion Efficiency of Perovskite/Silicon Tandem Solar Cells with Reduced Quantum Cutting Efficiency above Quantum Cutting Bandgap S10

Power Conversion Efficiency of 2T Tandem Solar Cells with Arbitrary Band Gaps under AM1.5G Illumination with Top Cell Thinning S11

Power Conversion Efficiency of Perovskite/Silicon Tandem Solar Cells with Quantum Cutting Layers under AM1.5G Illumination with Top Cell Thinning S12

References S13

Detailed Balance Analysis Method

The detailed balance limit of the tandem solar cell was calculated following the procedure laid out by Kirchartz *et al.*[1] The calculations were performed for a range of quantum cutting layer bandgaps from 2.53 to 4.5 eV ($E_{g,QC}$), top cell bandgaps from 0.5 to 2.5 eV ($E_{g,top}$), and bottom cell bandgaps from 0.5 to 2.5 eV ($E_{g,bot}$). The calculations were performed for both the AM 1.5G and AM 0 reference spectra ($\phi_{AM1.5G}$ or $\phi_{AM\,0}$) in units of photons·cm^{-2}·s^{-1}·eV^{-1}. For the sake of brevity, this explanation will only use the AM 1.5G ($\phi_{AM1.5G}$)

First, the quantum cutting layer was taken into account by assuming 100% absorption of the incident solar flux with energies above the $E_{g,QC}$ and 200% PLQY emission at 986 nm with a full width half max (FWHM) of 50 nm. The number of photons absorbed by the quantum cutting layer (α_{QC}) was determined by integrating the solar spectrum above the bandgap:

$$\alpha_{QC} = \int_{E_{g,QC}}^{\infty} \phi_{AM1.5G} dE$$

The values of $E_{g,QC}$ and α_{QC} were used to modify the solar spectrum incident on the underlying solar cell, ϕ_{inc}, where the incident photon flux at energies above $E_{g,QC}$ was 0 and the quantum cutting emission was added as a Gaussian peak.

$$\phi_{inc}(E) = \begin{cases} 0 \; if \; E > E_{g,QC} \\ \phi_{AM1.5G}(E) + A \cdot e^{\left(-\frac{(E-E_c)^2}{2\sigma^2}\right)} if \; E < E_{g,QC} \end{cases}$$

Next, the short-circuit current densities of the top and bottom cells ($J_{sc,top}$ and $J_{sc,bot}$, respectively) were calculated from

$$J_{sc,top} = q \int_{E_{g,top}}^{E_{g,QC}} \phi_{inc}(E) dE$$

$$J_{sc,bot} = q \int_{E_{g,bot}}^{E_{g,top}} \phi_{inc}(E) dE$$

where q is the elementary charge. The saturation current density (J_0) for each subcell was calculated according to

$$J_{0,top} = \int_{E_{g,top}}^{\infty} \phi_{bb}(E, T = 300\ K)\ dE$$

$$J_{0,bot} = \int_{E_{g,bot}}^{\infty} \phi_{bb}(E, T = 300\ K)\ dE$$

$$\phi_{bb}(E) = \frac{2\pi E^2}{h^3 c^2} \frac{1}{\left(e^{E/kT} - 1\right)}$$

where $\phi_{bb}(E, T = 300\ K)$ is the temperature dependent blackbody radiation of the two subcells at a temperature (T) of 300 K, h is Planck's constant, c is the speed of light, and k is Boltzmann's constant. Note that this saturation current density calculation neglects optical coupling effects such as absorption of emitted radiation by the bottom cell onto the top cell.

The current-voltage (JV) curve under illumination at the detailed balance limit could be calculated as

$$J_{top}(V) = J_{0,top}\left[e^{\left(qV/kT\right)} - 1\right] - J_{sc,top}$$

$$J_{bot}(V) = J_{0,bot}\left[e^{\left(qV/kT\right)} - 1\right] - J_{sc,bot}$$

where k is the Boltzmann constant. This results in an open-circuit voltage (V_{OC}) for each subcell of

$$V_{OC,top} = \frac{kT}{q} \ln\left(\frac{J_{sc,top}}{J_{0,top}} + 1\right)$$

$$V_{OC,bot} = \frac{kT}{q} \ln\left(\frac{J_{sc,bot}}{J_{0,bot}} + 1\right)$$

The power (P) for each subcell in the tandem was calculated

$$P_{top}(V) = -J_{top}(V) \cdot V$$

$$P_{bot}(V) = -J_{bot}(V) \cdot V$$

with the maximum power being used to calculate the maximum subcell efficiency (η)

$$\eta_{top} = \frac{\max(P_{top})}{\int_0^{\infty} E\phi_{AM1.5G}(E)dE}$$

$$\eta_{bot} = \frac{\max(P_{bot})}{\int_0^{\infty} E\phi_{AM1.5G}(E)dE}$$

By calculating these parameters for a given $E_{g,QC}$, $E_{g,top}$, and $E_{g,bot}$, the overall single junction, two-terminal (2T) tandem, and four-terminal (4T) tandem solar cell efficiencies could be calculated. The single junction efficiency was taken as the top cell efficiency ignoring the bottom cell. For a 4T configuration, the overall tandem efficiency (η_{4T}) was

$$\eta_{4T} = \eta_{top} + \eta_{bot}$$

since the two subcells do not electrically interact and optical coupling was neglected for these calculations.

The 2T configuration, on the other hand, was slightly more complicated since the top and bottom cells must additionally match currents. In order to determine the maximum power point of the 2T tandem, the voltage-current curve (as opposed to the current-voltage curve) of the two cells in series was calculated

$$V_{top}(J) = \frac{kT}{q} ln\left(\frac{J + J_{sc,top}}{J_{0,top}} + 1\right)$$

$$V_{bot}(J) = \frac{kT}{q} ln\left(\frac{J + J_{sc,bot}}{J_{0,bot}} + 1\right)$$

$$V_{2T}(J) = V_{top}(J) + V_{bot}(J)$$

and the overall maximum power point was found

$$P_{2T}(J) = -V_{2T}(J) \cdot J$$

resulting in a 2T tandem efficiency of

$$\eta_{2T} = \frac{\max(P_{2T})}{\int_0^\infty E\phi_{AM1.5G}(E)dE}$$

Modified Spectrum after Quantum Cutter under AM0 Illumination

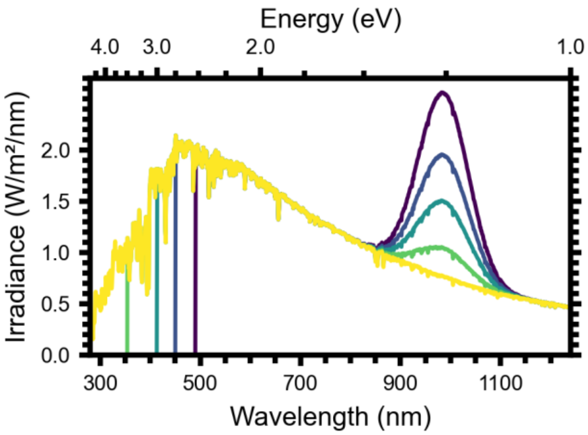

Figure S1. AM0 spectrum after modification by a quantum cutting layer with QC_{gap} ranging from 4.5 (yellow) to 2.53 eV (purple).

Efficiency Limit of Single Junction Solar Cell with Quantum Cutting under AM0 Illumination

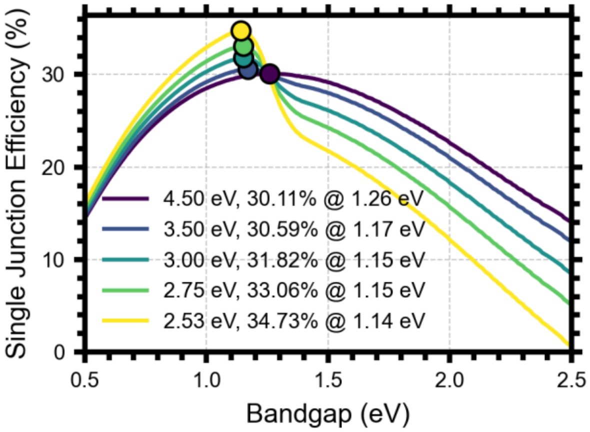

Figure S2. Single junction efficiency limit under AM0 with varying quantum cutting band gap.

Power Conversion Efficiency of Tandem Solar Cells with Arbitrary Band Gaps under AM0 Illumination

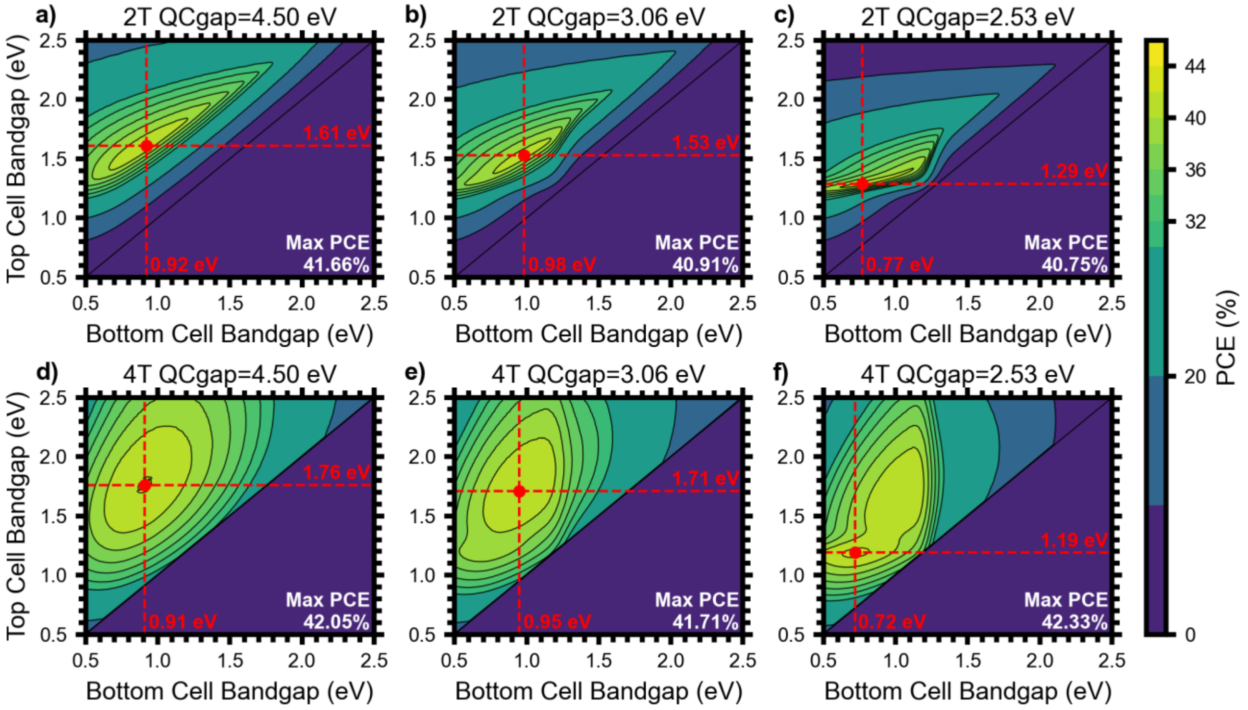

Figure S3. Contour plots of the power conversion efficiency as a function of the bottom (x-axis) and top cell (y-axis) bandgaps for a two-junction tandem solar cell in 2T (**a-c**) or 4T (**d-f**) configurations. Plots are varied for no quantum cutting layer (**a, d**), Yb:$CsPbCl_3$ (**b, e**), or mixed Yb:$CsPb(Cl_xBr_{1-x})_3$ with quantum cutting bandgap of 2.53 eV (**c, f**).

The trends seen here in the optimal top and bottom cells largely follow the same trends as the AM1.5G irradiance scenario in which the optimal bandgaps shift to lower energy. However, the shifts are more dramatic since there is a larger amount of UV light present in the AM0 spectrum, resulting in a dramatic shift for the lowest value of the QC_{gap}, in which the optimal combination utilizes a 1.2-1.3 eV top cell with a 0.72-0.77 eV bottom cell. Similar to the trends in the AM1.5G scenario, the PCE_{max} does not change substantially for either architecture.

Power Conversion Efficiency of Perovskite/Silicon Tandem Solar Cells with Quantum Cutting Layers under AM0 Illumination

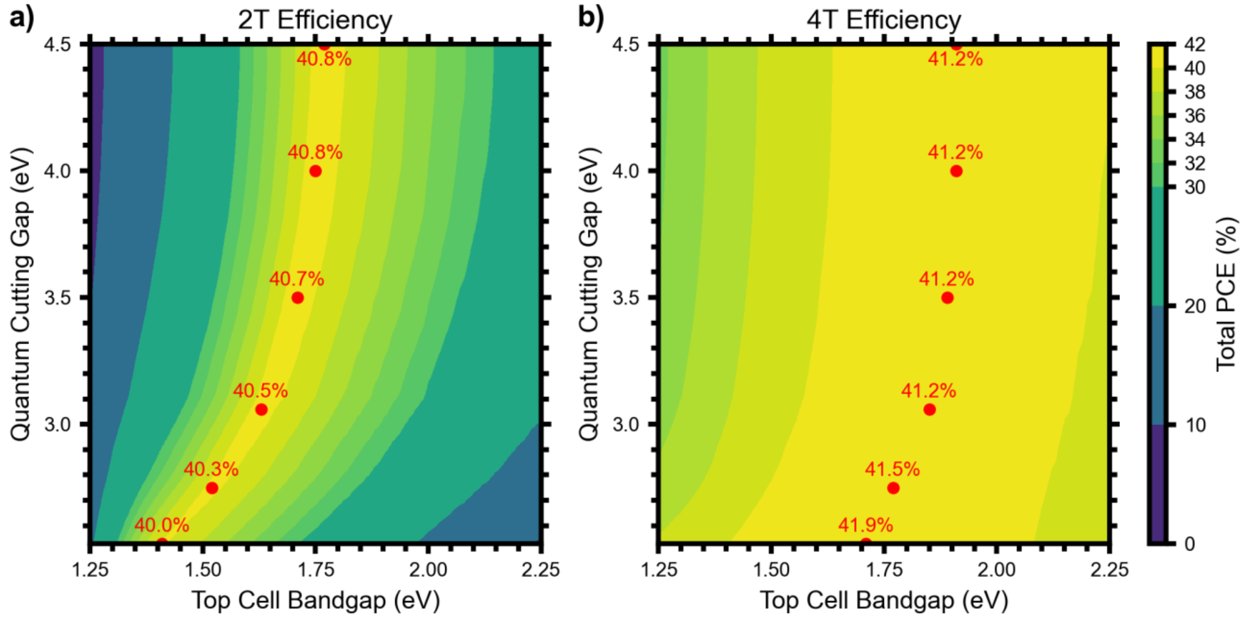

Figure S4. Power conversion efficiency for perovskite/silicon tandem solar cells with varying top cell bandgaps (x-axis) and quantum cutting bandgaps (y-axis) for the 2T (**a**) and 4T (**b**) architectures.

Similar to the trends in optimal bandgaps for the perovskite/silicon tandem solar cell under AM1.5G irradiation, the optimal top cell bandgap shifts to lower energy with decreasing QCgap. Under AM0, the shift for the 4T architecture is more pronounced since the top cell benefits from increasing the photovoltage harvested from the ~1.7 eV photons. Again, the PCE_{max} change is relatively minimal for both architectures, decreasing slightly for the 2T and increasing slightly for the 4T.

Power Conversion Efficiency of Perovskite/Silicon Tandem Solar Cells with Reduced Quantum Cutting Efficiency above Quantum Cutting Bandgap

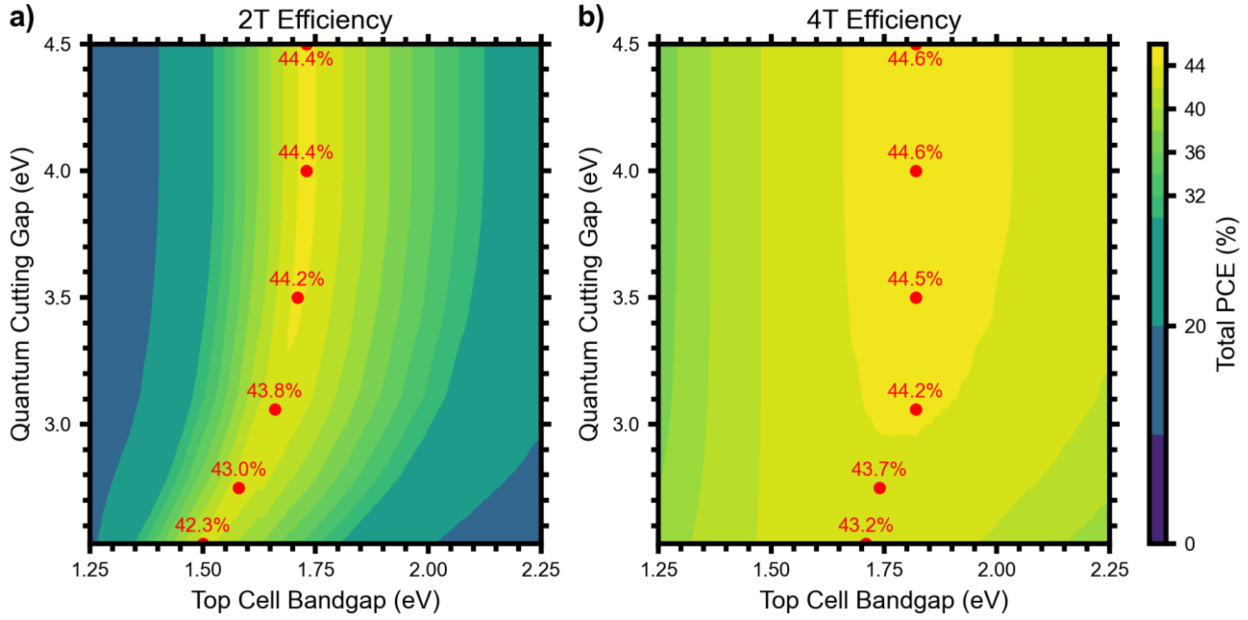

Figure S5. Power conversion efficiency for perovskite/silicon tandem solar cells with varying top cell bandgaps (x-axis) and quantum cutting bandgaps (y-axis) for the 2T (**a**) and 4T (**b**) architectures with 150% quantum efficiency above the quantum cutting bandgap, as opposed to the theoretical limit of 200% quantum efficiency modeled in the main text.

Power Conversion Efficiency of 2T Tandem Solar Cells with Arbitrary Band Gaps under AM1.5G Illumination with Top Cell Thinning

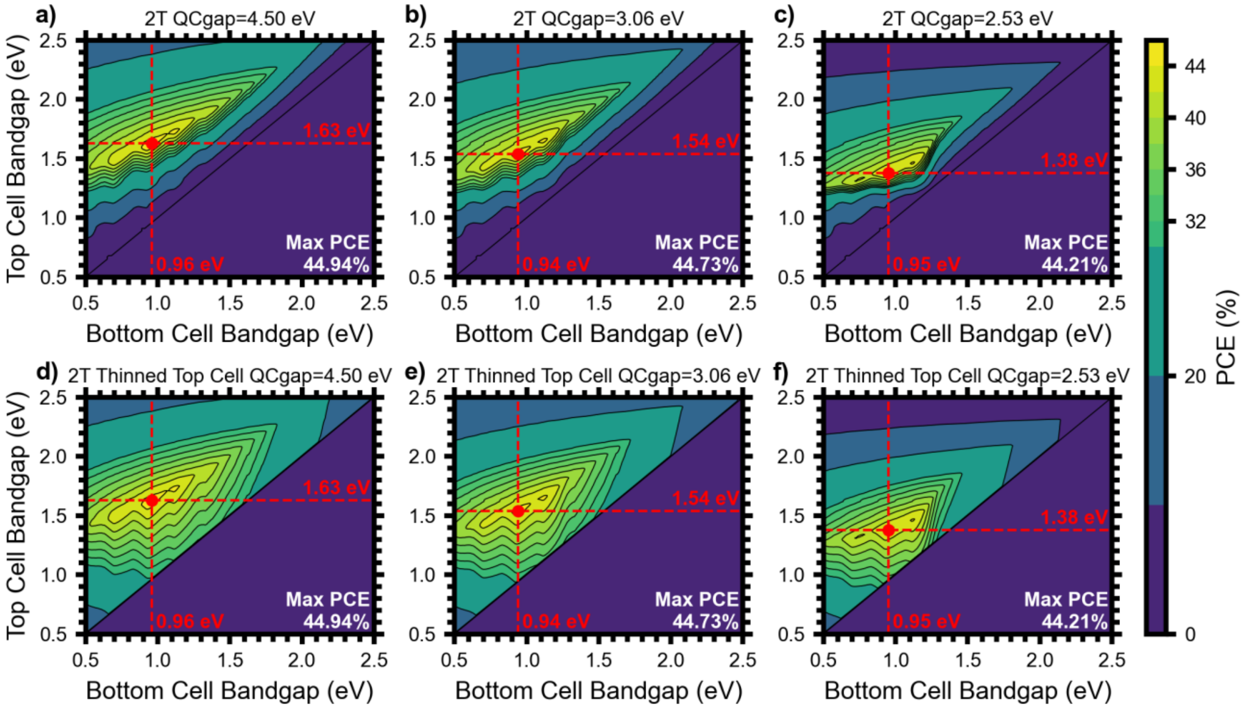

Figure S6. Contour plots of the power conversion efficiency as a function of the bottom (x-axis) and top cell (y-axis) bandgaps for a 2T configuration with a step function 100% absorption above the bandgap (**a-c**, reproduced from **Figure 2a-c** in the main text) or varied top cell absorption to allow optimal current matching (**d-f**). Plots are varied for no quantum cutting layer (**a, d**), Yb:$CsPbCl_3$ (**b, e**), or mixed Yb:$CsPb(Cl_xBr_{1-x})_3$ with quantum cutting bandgap of 2.53 eV (**c, f**).

The calculated efficiency limit of 2T tandem solar cells is limited in many cases by the assumed 100% absorption above the top cell bandgap. As a result, the short circuit current of the bottom cell limits the performance of the tandem solar cell (**Figure S5a-c**). In a practical tandem solar cell, the top cell could be thinned to allow for some transmission of the above bandgap light (**Figure S5d-f**). This change in assumption does not increase the absolute maximum PCE but does increase the PCE limit for many combinations of top and bottom cell bandgaps, creating a broader PCE limit plateau.

Power Conversion Efficiency of Perovskite/Silicon Tandem Solar Cells with Quantum Cutting Layers under AM1.5G Illumination with Top Cell Thinning

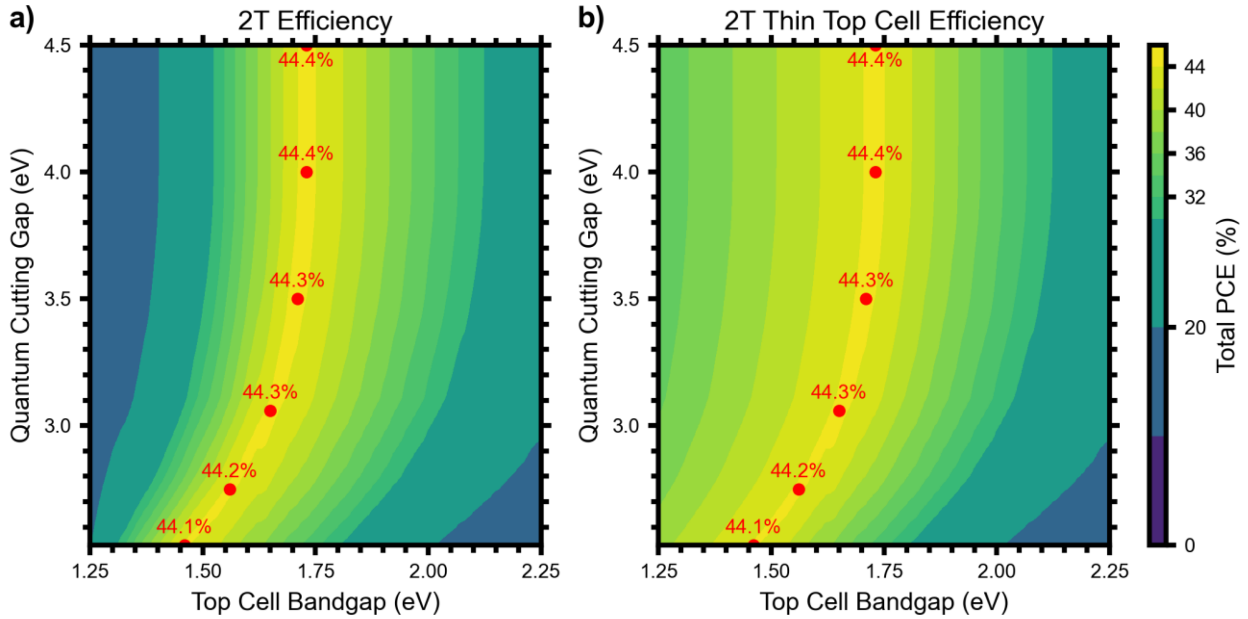

Figure S7. Power conversion efficiency for perovskite/silicon tandem solar cells with varying top cell bandgaps (x-axis) and quantum cutting bandgaps (y-axis) for the 2T with 100% absorbance above bandgap (**a**, reproduced from **Figure 3a** in the main text) and thinned top cell with optimal current matching with the silicon bottom cell (**b**).

The calculated efficiency limit of 2T perovskite/silicon tandem solar cells is limited in many cases by the assumed 100% absorption above the top cell bandgap. As a result, the short circuit current of the bottom cell limits the performance of the tandem solar cell for values of the top cell bandgap below the optimum value (left hand side of **Figure S6a**). In a practical tandem solar cell, the top cell could be thinned to allow for some transmission of the above bandgap light. This does not change the optimum value of the top cell bandgap or the maximum efficiency (**Figure S6b**, red points) but does increase the PCE limit for values of the top cell bandgap less than the optimum value (left hand side of **Figure S6a**). The efficiency when the top cell bandgap limits the current and efficiency of the 2T tandem solar cell (right hand side of **Figure S6a and S6b**) is unchanged.

References

(1) Kirchartz, T.; Rau, U. What Makes a Good Solar Cell? *Adv. Energy Mater.* **2018**, *8* (28), 1703385. https://doi.org/10.1002/aenm.201703385.
